# Community's Perception and Attitude towards People with Epilepsy in Ethiopia

**DOI:** 10.1155/2019/4681958

**Published:** 2019-11-07

**Authors:** Wubalem Fekadu, Tesfa Mekonen, Shemelash Bitew, Tefera Chanie Mekonnen, Melak Menberu, Seble Shewangizaw

**Affiliations:** ^1^Psychiatry Department, College of Medicine and Health Sciences, Bahir Dar University, Bahir Dar, Ethiopia; ^2^Psychiatry Department, College of Health Sciences, Addis Ababa University, Addis Ababa, Ethiopia; ^3^School of Public Health, College of Medicine and Health Sciences, Wolaita Sodo University, Wolaita Sodo, Ethiopia; ^4^School of Public Health, College of Medicine and Health Sciences, Wollo University, Dessie, Ethiopia

## Abstract

**Introduction:**

Most people with epilepsy suffer from a dual burden. In one hand, they struggle with the symptoms and disabilities on the other hand from misconceptions and stigma associated with it. But there are no recent studies which assess the community's perception and attitude.

**Objective:**

To assess the perception and attitude of the community towards people with epilepsy and identify associated factors.

**Methods:**

A community-based cross-sectional study was conducted in South Ethiopia from a total of 701 participants. Data were collected with face to face interview using a structured questionnaire developed based on the Health Belief Model (HBM). Data were presented with frequencies, tables, and figures. Univariate and multivariable logistic regression was done to identify significantly important variables. The presence of association was presented by odds ratio and 95% confidence interval. Ethical clearance was obtained from Wolaita Sodo University.

**Results:**

The most frequently mentioned perceived causes for epilepsy were stress (91%), substance use (61.8%), and bad spirit (49.8%) while loss of consciousness and falling (80.7%) and sleep problems (78%) were considered symptoms of epilepsy. Only 13.1% of the participants think that they may be susceptible for epilepsy. Six hundred sixty (94.2%) participants will not employ a person with epilepsy while only 47 (6.7%) of the participants will allow a family member to marry a person with epilepsy. In multivariable analysis, understanding the illness as a medical problem was associated with perceived susceptibility and perceived benefit of modern treatment was significantly associated with having a current medical problem.

**Conclusions:**

The knowledge about the cause, possible susceptibility, better treatment options, and attitude of the participants were similar to other low-income settings. The negative attitude was high and multidimensional. All stakeholders must work to increase awareness about the cause, symptoms, and treatment options for epilepsy and to decrease the negative attitude of the community.

## 1. Introduction

Epilepsy is a chronic neurological problem mainly characterized by tonic-clonic seizures and other associated symptoms. The global prevalence ranges from 2.7 to 17.6 per 1000 while the incidence ranges from 2.2 to 41.0 per 100 population. More than 80% of the global cases are in developing countries. The difference was attributed to access to health care, regional environmental exposures, or socioeconomic status. The illness is much higher in children and older people [1, 2].

Though nearly four-fifth of people with epilepsy are in low-income settings, the treatment gap reaches up to 90% [3]. The treatment gap is attributed to lack of trained health professionals, treatment cost, availability of medicines, residence (urban/rural), and attitude of patients, family, and the larger community [4, 5].

The community's perception and attitude are important determinants in epilepsy care because it affects the help-seeking behavior of people with epilepsy, their family members, and policymakers. The perception of the community is different in high-income and low-income settings. The explanatory model in high-income settings is mainly scientific while in low-income countries the explanations are mainly to traditional and religious causes [4, 6–9].

In Austria, there was 10% negative attitude towards people with epilepsy in the general population which was significantly associated with gender and economic status [10] while in Croatia, 7% of the participants would object if their child played with a child with epilepsy, and 76% believed that a child with epilepsy could succeed as well as a child without epilepsy. The better attitude was associated with knowing someone with epilepsy and/or witnessing a seizure [11].

In Greece, 19% consider epilepsy as a type of mental retardation, and 15% believed it is a type of insanity, while 5.2% considered it a supernatural phenomenon. Seventy-seven percent considered epilepsy a curable disease, 57.5% believed that the risk of inheriting it is very high, and 45.4% rejected marriage to the patient with epilepsy [12]. The same was true in Jordan where less than 50% accepts letting their children play with children with epilepsy or employ people with epilepsy. Nine percent had negative attitudes and believed that patients with epilepsy are insane, and 88.5% objects the marriage of people with epilepsy to their sons or daughters [13].

In Thailand, out of 1581 research participants, 80.8% were familiar with the word epilepsy. The main reason given for avoiding helping a seizure victim was lack of proper knowledge. The negative attitude was predicted by low educational level, unfamiliarity with epilepsy, and the misconception that epilepsy is a form of insanity [14]. The Tanzanian case was different, where 46.7% of the participants think that epilepsy was due to supernatural causes and 65.3% think that people with epilepsy should not attend school or go to work [15]. In Ethiopia, a study on people with epilepsy and their caregivers shows the proportion of perceived stigma was 81%, and it was associated with occupation and frequency of seizure episodes [16].

The studies in Ethiopia focused on the perceived and experienced stigma of people with epilepsy and their family members [16–18] not the general community which is the main player in stigma and discrimination. The aim of the current study is to assess the community's perception and attitude in a relatively large sample size in the semiurban population. This result will benefit researchers, policymakers, and other stakeholders in most low-income countries.

## 2. Methods

### 2.1. Study Design and Settings

A community-based cross-sectional study was conducted in Wolaita Sodo town which is 387 km south of the capital Addis Ababa. The town had a total population of 110,660. There are many private and public health institutions where people with epilepsy can get medical help. This availability of care is the reason to choose the setting Wolaita Sodo.

### 2.2. Sample Size Determination and Participants

The sample size was calculated with EPI-info [19] considering the following assumptions:

- Confidence level = 95%
- *α* = 0.05
- Proportion = 65% of residents will have negative perception [20]
- *d* = 0.05
- Design effect = 2 because of the clustering [21]
- Possible nonresponse rate = 5%

**Figure 1:**
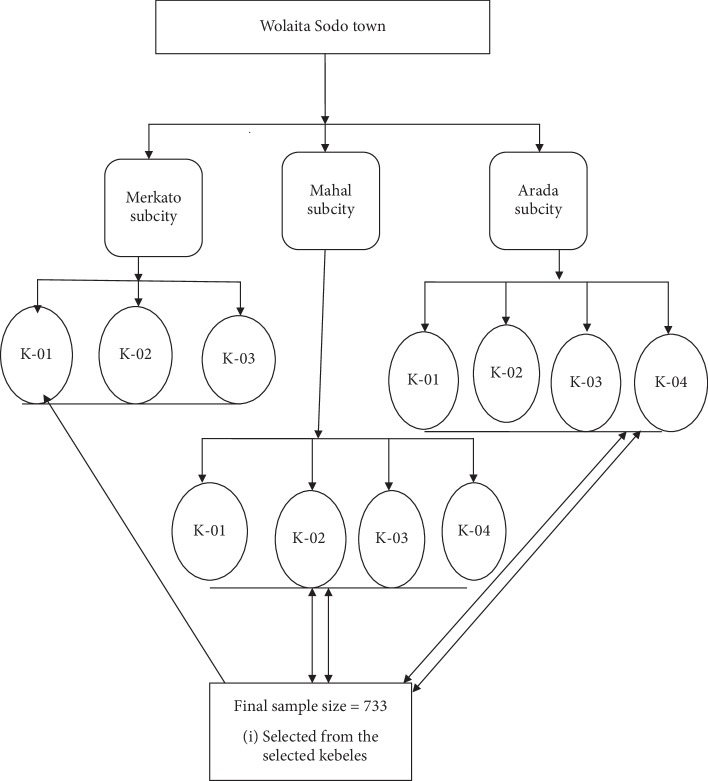
Participant recruitment procedure.

Participants were available heads (husband or wife) of the selected households (*n* = 701) from five randomly selected kebeles (lowest administrative units). The kebeles were selected from all three subcities of the town considering the size of the population (Figure 1 details the participant recruitment process).

### 2.3. Study Variables

Communities' perception about the cause, treatment, and prevention of epilepsy was the dependent variable while the independent variables include sociodemographic factors (age, sex, marital status, education, and occupation), family history, knowledge about the illness, and attitude towards people with epilepsy.

### 2.4. Data Collection Tools, Procedures, and Analysis

Semistructured interview was developed based on a Health Belief Model (HBM) and translated into Amharic (the official language of the town). The questionnaire had six parts: sociodemographic factors, perceived cause and symptoms of epilepsy, perceived seriousness, perceived benefit of treatment, perceived susceptibility for epilepsy, and attitude towards people with epilepsy. We (three experienced mental and public health researchers) have done forward and backward translation to maintain consistency. Each researcher has done the translation independently and console the differences together.

We assessed the participant's awareness about the cause and symptoms of epilepsy and attitude and practice towards people with epilepsy with vignette-based diagnosis. The vignettes are developed based on possible scenarios of people with tonic-clonic epilepsy (annex 1). Attitude questions include work opportunity, marital prospects, chance of education, severity of the disease, and chance of management by modern medicine.

Data were collected by five trained diploma nurses and supervised by two psychiatry nurses. A two-day training was given for data collectors and supervisors. The instrument was piloted in the nearby town. The data collectors were supervised daily, and the filled questionnaires were checked daily by the supervisors and first author for completeness and consistency. Data were entered to Epi data 3.02 [22], and analysis was done with SPSS-21 [23]. We present the data with frequency numbers, tables, and figures. A value less than 0.05 will be considered statistically significant. Univariate and multivariable logistic regression was done to identify significantly important variables. The presence of association was presented by odds ratio and 95% confidence interval [24]. Ethical clearance was obtained from the ethical review committee of Wolaita Sodo University (CHS 02/15). Written consent was taken from each participant.

## 3. Results

Three hundred sixty-one (51.5%) participants were males, and 30.4% were in the age group of 28-32. Six hundred fifty-seven (93.7%) of them were married, and 46.2% of the participants had certificate and above by educational status. One-third of the participants were government employees, and their estimated monthly income ranges from 400 to 15,000 birr (Table 1).

### 3.1. Clinical Characteristics of the Participants

From the total of 701 participants, 29 (4.14%) had a reported known history of neuropsychiatric illness. Sixty-five (9.3%) participants had a reported family history of neuropsychiatric illnesses while seventy-eight (11.1%) participants had a history of sharing a household with a person with known epilepsy.

### 3.2. Explanatory Model for Epilepsy

#### 3.2.1. Causes and Symptoms of Epilepsy

Stress was mentioned by 638 (91%) of the respondents as a cause of epilepsy followed by substance use, which was mentioned by 433 (61.8%) of the participants (table). Loss of consciousness and falling were mentioned by 80.7% of the study participants as a symptom of epilepsy, followed by sleep problems which were mentioned by 78% of the participants (Table 2).

#### 3.2.2. Perceived Susceptibility

Only 92 (13.1%) of the participants think that they are susceptible to epilepsy. This susceptibility was associated with understanding the illness as a medical problem in multivariable analysis (Table 3).

#### 3.2.3. Perceived Seriousness

Based on case vignettes, 627 (89.4%) of the participants consider epilepsy a serious illness which results in death and disability while the remaining 74 (10.6%) consider it a benign illness (Table 4). More than half (56.9%) of the participants think that epilepsy is preventable by different means: reducing stress, increasing social interaction, having a frequent medical checkup, change stressful workplace, and praying. Participants who consider it not preventable put the following reasons: epilepsy is the result of God's will, the cause is unknown, and the onset is sudden. The common impacts of epilepsy mentioned by the participants were problems in the functioning and susceptibility for accident and death.

#### 3.2.4. Perceived Benefit

Most (87%) of the participants report that modern treatment is helpful for mental illness. Among them, 45.2% of the participants think that treatment results in complete recovery and the remaining 41.3% of them report that modern treatment can help peoples to proceed with their work and to hinder the symptoms temporarily. Participants who consider modern treatment as not helpful consider traditional treatment and religious help as better options.

In multivariable analysis, perceived benefit of modern treatment was significantly associated with having a current medical problem (Table 5).

#### 3.2.5. Attitude towards People with Epilepsy

Six hundred sixty (94.2%) of the participants will not employ a person with epilepsy while only 47 (6.7%) of the participants will allow a family member to marry a person with epilepsy (Table 4).

## 4. Discussion

There were many scientific advances in the treatment of epilepsy in recent years, but the treatment gap is still high especially in low-income settings which is mainly attributed to poor perception and bad attitude towards people with epilepsy.

The Ethiopian case is the same; though the health care coverage has increased a lot in recent years, the treatment gap is still very high. This may be attributable to different factors. Community's low awareness and negative attitude towards people with epilepsy are two of the factors which played a negative role for this high treatment gap. This paper is one its kind which conceptualizes the public awareness and attitude about epilepsy in a relatively large sample size in recent years.

The perceived cause for epilepsy in this study is fairly different from previous studies in low-income countries where participants mention biological causes as the main cause of the illness [14, 15, 25, 26]. This was not the case in other studies where more than two-thirds of the people attribute the illness for spiritual causes. But nearly half of our participants also mention spiritual causes. This may be due to the living place of the participants (the participants in this study are living in the urban and semiurban areas) where people in urban and semiurban settings have access to information. The other explanation may be the way the questionnaire was framed; in our case, the participants could mention multiple cases.

The loss of consciousness, falling, and sleep problems were the common symptoms mentioned by the participants. This was consistent across studies in both high- and low-income countries. The main reason for this agreement may be because of the focus of the studies on tonic-clonic seizure which is characterized by loss of consciousness and falling [1, 11, 12].

Perceived susceptibility is considered an important predictor of stigma. If a person thinks he/she is susceptible for any illness, the chance to stigmatize someone is lesser and the chance to sympathize will be higher. But perceived susceptibility was not the focus of epilepsy studies. In the current study, only 13% of the participants perceive as they may be susceptible to the illness. It was significantly associated with age and the participants overall understanding about epilepsy as a disease.

Most of the participants consider epilepsy a serious illness which results in death or disability. The result is consistent with previous studies [13, 27, 28]. More than half of them think that it is preventable which is much higher than previous studies. This supports the perceived cause of the illness; for example, participants think that reducing stress will reduce the chance of developing the incidence of epilepsy.

Eighty-seven percent of the participants consider modern medical treatment as a better treatment option for epilepsy which is contrary to the treatment gap, i.e., nearly 90% [3, 4]. The possible explanations for these can be the living place of the participants and possible social desirability bias. The multivariable analysis indicated that perceived modern treatment was significantly associated with having a current medical problem other than epilepsy. This may be because these people with other medical problems may have higher health literacy because they may visit health institutions frequently [29].

The perception about epilepsy and people with epilepsy will affect their attitude towards people with epilepsy. The attitude of the community towards this people with epilepsy is negative in both developed and developing countries. The same was true in our study where most of the participants were not willing to recruit a person with epilepsy and will not allow a family member to marry a person with epilepsy [10, 30].

## 5. Limitations

This study is done in a relatively large sample with a pretested questionnaire, but it has its own limitations. The first limitation is related to the social desirability in some sections such as the perceived benefit of modern treatment; as we are from modern health institutions, they may tend to report the perceived benefit of medicines. The second limitation is related to the place of residence; our study settings were urban and semiurban areas, so it may not be generalizable for the rural settings.

## 6. Conclusions

The knowledge about the cause, possible susceptibility, better treatment options, and attitude of the participants were similar to other low-income settings. The negative perception and attitude may increase stigma, preclude help-seeking, and increase the burden of epilepsy. So all stakeholders must work to increase awareness about the cause, symptoms, and treatment options for epilepsy and to decrease the negative attitude of the community.

## Figures and Tables

**Table 1:** Sociodemographic characteristics of the participants (*n* = 701).

Variable		Frequency (%)
Age	23-27	121 (17.3)
	28-32	213 (30.4)
	33-37	101 (14.4)
	38-42	152 (21.7)
	≥43	114 (16.3)

Religion	Protestant	438 (62.5)
	Orthodox	201 (28.7)
	Muslim	34 (4.9)
	Catholic	28 (4)

Marital status	Single	13 (1.9)
	Divorced	4 (0.6)
	Widowed	5 (0.7)
	Separated	22 (3.1)
	Married	657 (93.7)

Educational status	Illiterate	17 (2.4)
	Primary school	93 (13.3)
	Secondary school	267 (38.1)
	Certificate and above	324 (46.2)

Occupational	Government	246 (35.1)
	Private employ	140 (20)
	Merchant	126 (18)
	House wife	126 (18)
	Daily worker	44 (6.3)
	Student	19 (2.7)

**Table 2:** Participant's perceived cause of epilepsy (*n* = 701).

Cause of epilepsy	Yes/no	Frequency (%)
Poverty	No	423 (60.3)
	Yes	278 (39.7)

Substance use	No	268 (38.2)
	Yes	433 (61.8)

Bad sprit	No	352 (50.2)
	Yes	349 (49.8)

Possession	No	492 (70.2)
	Yes	209 (29.8)

Genetics	No	399 (56.92)
	Yes	302 (43.08)

God's will	No	544 (77.6)
	Yes	157 (22.4)

Social isolation	No	455 (64.9)
	Yes	246 (35.1)

Stress	No	63 (9)
	Yes	638 (91)

Sudden event	No	559 (79.74)
	Yes	142 (20.26)

Medical illness	No	528 (75.3)
	Yes	173 (24.7)

Have no idea		39 (5.56)

**Table 3:** Factors associated with perceived susceptibility.

Variables		Perceived susceptibility		COR (95% CI)	AOR (95% CI)
		Yes	No		
Age	23-27	10	111	1	1
	28-32	34	179	2.11 (1.002, 4.44)	
	33-37	15	86	1.94 (0.83, 4.52)	
	38-42	16	136	1.31 (0.57, 2.99)	
	>42	17	97	1.95 (0.85, 4.45)	

Consider the symptoms illness	No	19	55	2.62 (1.47, 4.66)	**1.8 (1.4, 2.5)**
	Yes	73	554	1	**1**

**Table 4:** Participant's perceived impacts and attitude towards people with epilepsy.

Variable	Yes/no	Frequency (%)
*Impacts of epilepsy*		
Unable to work	Yes	464 (66.2)
	No	237 (33.8)
Economic problem	Yes	445 (63.5)
	No	256 (36.5)
Social dysfunction	Yes	447 (63.8)
	No	254 (36.2)
Susceptibility	Yes	462 (65.9)
	No	239 (34.1)
Death	Yes	501 (71.5)
	No	200 (28.5)
*Attitude towards people with epilepsy*		
Will you employ a person with epilepsy?	No	660 (94.2)
	Yes	41 (5.8)
Will you allow family member to marry a person with epilepsy?	No	654 (93.3)
	Yes	47 (6.7)
Will you allow having in the same house with mentally ill?	No	430 (61.3)
	Yes	271 (38.7)
Will you allow having a neighbor with epilepsy?	No	256 (36.5)
	Yes	445 (63.5)
Do you think a person with epilepsy can learn, work?	No	351 (50.1)
	Yes	350 (49.9)
Do you think epilepsy is manageable?	No	203 (29)
	Yes	498 (71)

**Table 5:** Factors associated with perceived benefit of modern treatment for epilepsy.

Variables		Perceived benefit of treatment		COR (95% CI)	AOR (95% CI)
		Yes	No		
Occupation	Gov't employee	206	40	1	1
	Private employee	125	15	1.62 (0.86, 3.05)	
	Merchant	111	15	1.44 (0.76, 2.72)	
	House wife	114	12	1.85 (0.93, 3.66)	
	Daily worker	54	9	1.17 (0.53, 2.55)	

Consider the symptoms illness	No	39	35	1	1
	Yes	571	56	9.15 (5.3, 15.59)	

Current medical illness	No	159	142	1	**1**
	Yes	91	24	3.39 (2.05, 5.6)	**2.4 (1.39, 4.17)** ^∗^

## Data Availability

The data used to support the findings of this study are available from the corresponding author upon request.

## Additional Points

*Implications*. The outcome of the study had research and policy implications. Researchers need to assess the community's awareness and attitude towards epilepsy in any clinical outcomes while clinicians need to work on communities' attitude and awareness in delivering care for the people with epilepsy. The study had also policy implication by showing areas to increase awareness and work on negative attitudes towards people with epilepsy. *Data Sharing*. The data will be shared upon reasonable request.
